# Heterogeneity of the tumor immune microenvironment and its clinical relevance

**DOI:** 10.1186/s40164-022-00277-y

**Published:** 2022-04-23

**Authors:** Qingzhu Jia, Aoyun Wang, Yixiao Yuan, Bo Zhu, Haixia Long

**Affiliations:** 1 Institute of Cancer, Xinqiao Hospital, Army Military Medical University, Xinqiao Main Street, Chongqing, 400037 China; 2 Chongqing Key Laboratory of Immunotherapy, Xinqiao Hospital, Army Medical University, Chongqing, 400037 China; 3 Department of Thoracic Surgery, The Third Affiliated Hospital of Kunming Medical University, Kunming, 650118 China

**Keywords:** Tumor microenvironment, Heterogeneity, Immune checkpoint blockade, Immunotherapy

## Abstract

During the course of tumorigenesis and subsequent metastasis, malignant cells gradually diversify and become more heterogeneous. Consequently, the tumor mass might be infiltrated by diverse immune-related components, including the cytokine/chemokine environment, cytotoxic activity, or immunosuppressive elements. This immunological heterogeneity is universally presented spatially or varies temporally along with tumor evolution or therapeutic intervention across almost all solid tumors. The heterogeneity of anti-tumor immunity shows a profound association with the progression of disease and responsiveness to treatment, particularly in the realm of immunotherapy. Therefore, an accurate understanding of tumor immunological heterogeneity is essential for the development of effective therapies. Facilitated by multi-regional and -omics sequencing, single cell sequencing, and longitudinal liquid biopsy approaches, recent studies have demonstrated the potential to investigate the complexity of immunological heterogeneity of the tumors and its clinical relevance in immunotherapy. Here, we aimed to review the mechanism underlying the heterogeneity of the immune microenvironment. We also explored how clinical assessments of tumor heterogeneity might facilitate the development of more effective personalized therapies.

## Background

Tumorigenesis and cancer progression are dynamic evolutionary processes [1, 2]. Extensive studies on tumor evolution have enabled researchers to characterize the cancer genome diversification, track the spatial and longitudinal evolution of tumor cells, and explore the genetic determinants underlying these evolutionary events [3–10]. Accompanied by the evolution of tumor cells, the surrounding microenvironment can also be modulated through the interaction between genetic driving forces and environmental elements. On the contrary, there is a growing body of evidence supporting the impact of environmental elements on tumor evolution and progression [11–13]. Randomly generated mutations lead to the accumulation of subclones within the tumor cell population. Due to intrinsic variations among them, these subclonal tumor cells are forced to compete for growing cues and nutrition supplementation, allowing them to form spatially discrete niches in a limited lesion [14, 15]. In order to gain fitness in the surrounding environment, diverse subclonal tumor cells can actively modify the tumor microenvironment, including inducing pathologic angiogenesis for nutrient supply, disturbing the immune stimulatory/inhibitory checkpoint pathway to promote immune evasion, and remodeling the extracellular matrix to facilitate metastasis [1, 16]. Conversely, through a variety of mechanisms, environmental elements can instruct the evolution of tumor cells by selecting subclones with optimally adaptive phenotypes [11, 12]. These mutual modulations between tumor cells and the surrounding microenvironment could have a profound impact on the evolution and progression of cancer.

**Fig. 1:**
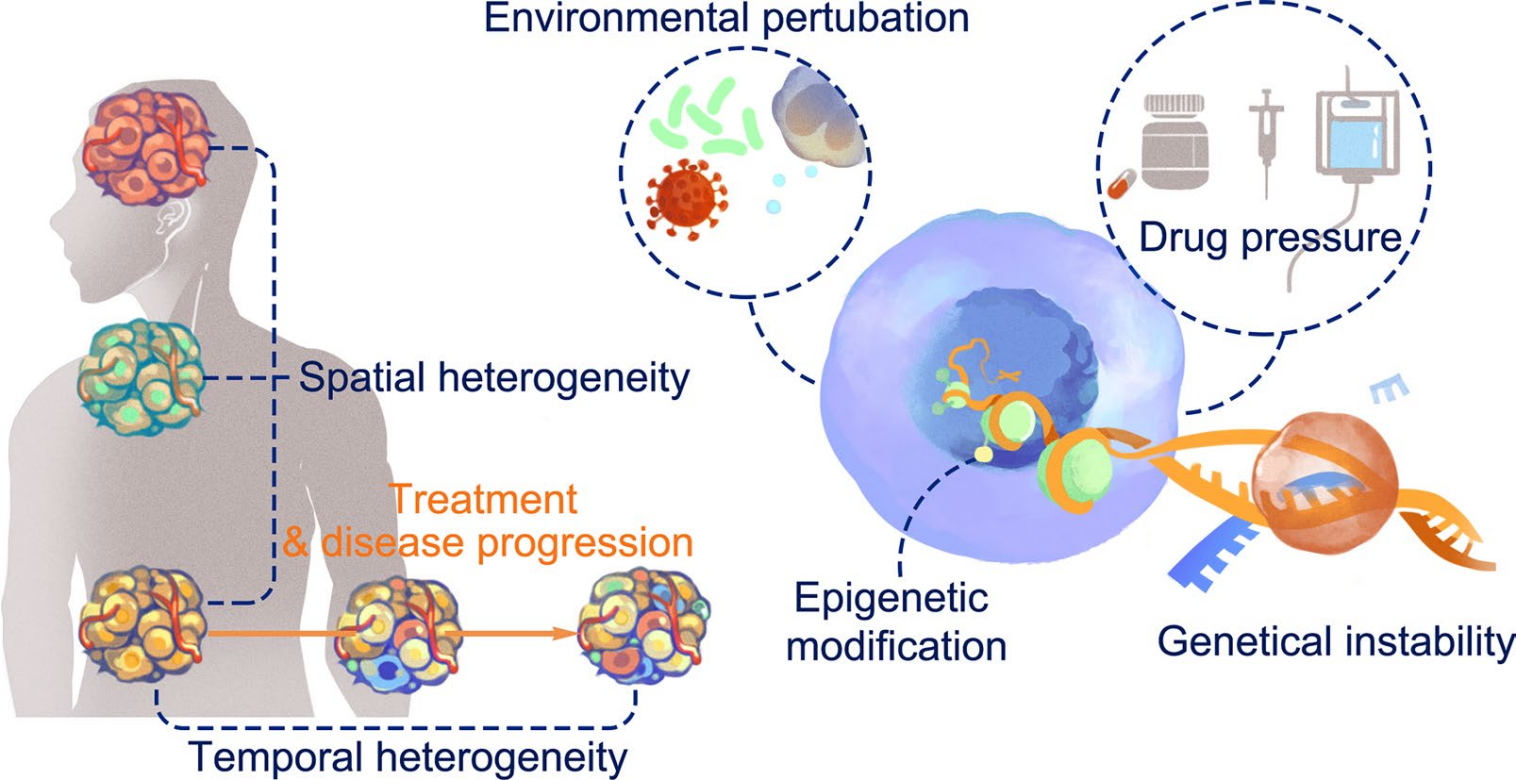
Origin and pattern of tumor immunological heterogeneity. Spatial heterogeneity represents an uneven localized immunological component within single tumor, or among intra-individually metastasized tumors. Temporal heterogeneity denotes the evolutionary dynamics of immunological components along the course of tumor progression, or in response to clinical intervention. Tumor immunological heterogeneity was originated from tumoral intrinsic event including genomic instability and epigenetic modification, or originated from extrinsic events such as environmental perturbations or therapeutic pressure

**Fig. 2:**
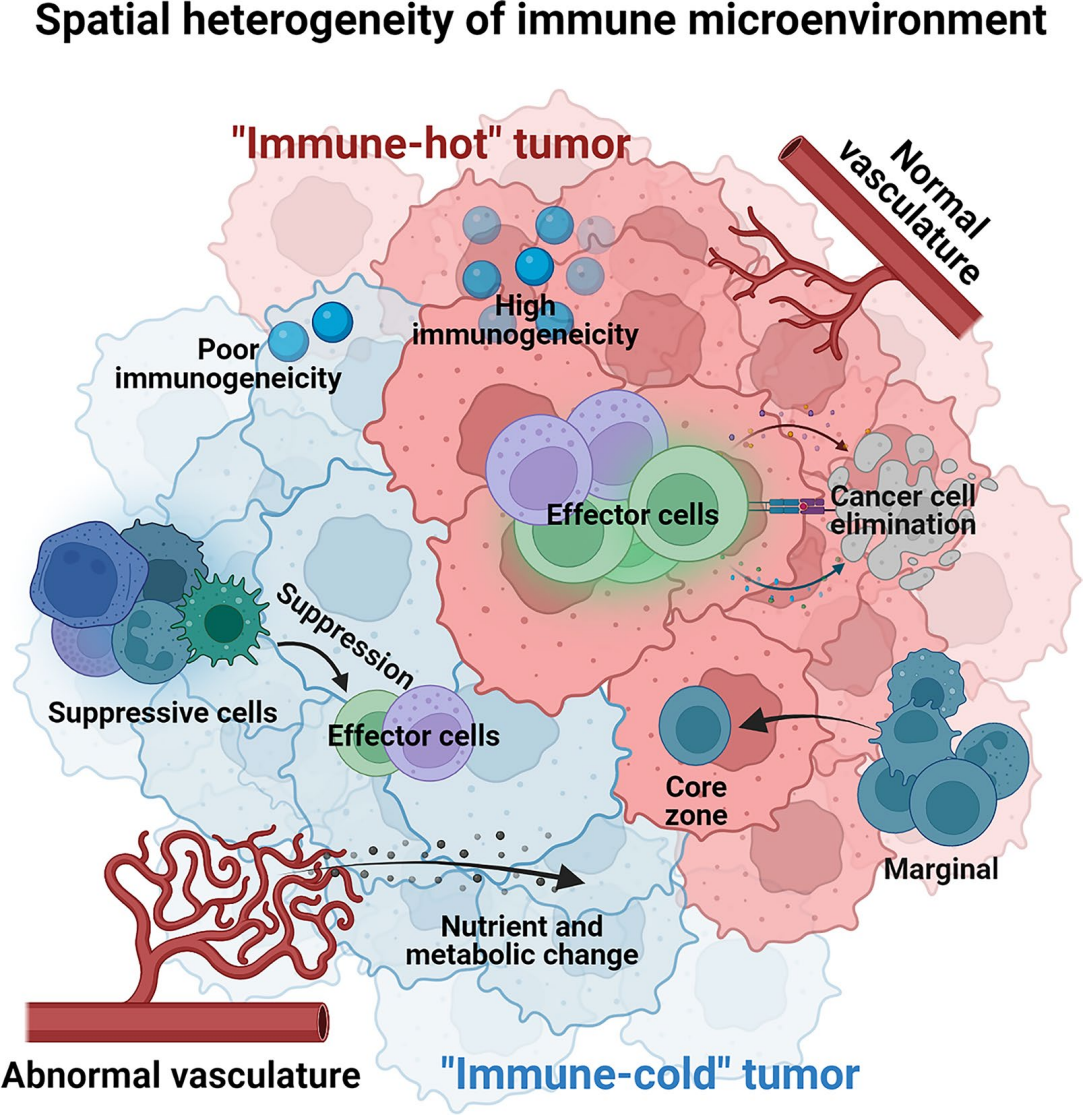
Spatial heterogeneity of immune microenvironment. Tumor immune microenvironment was reported that can be broadly divided into immune-hot or -cold based on whether it favor an effective anti-tumor immune response or not. Representative traits of a heterogeneous immune microenvironment including the spectrum of neoantigen, the infiltration of immunological suppressive cells and effector cells, the status of vasculature, the milieu of cytokine/nutrient and metabolic program

**Fig. 3:**
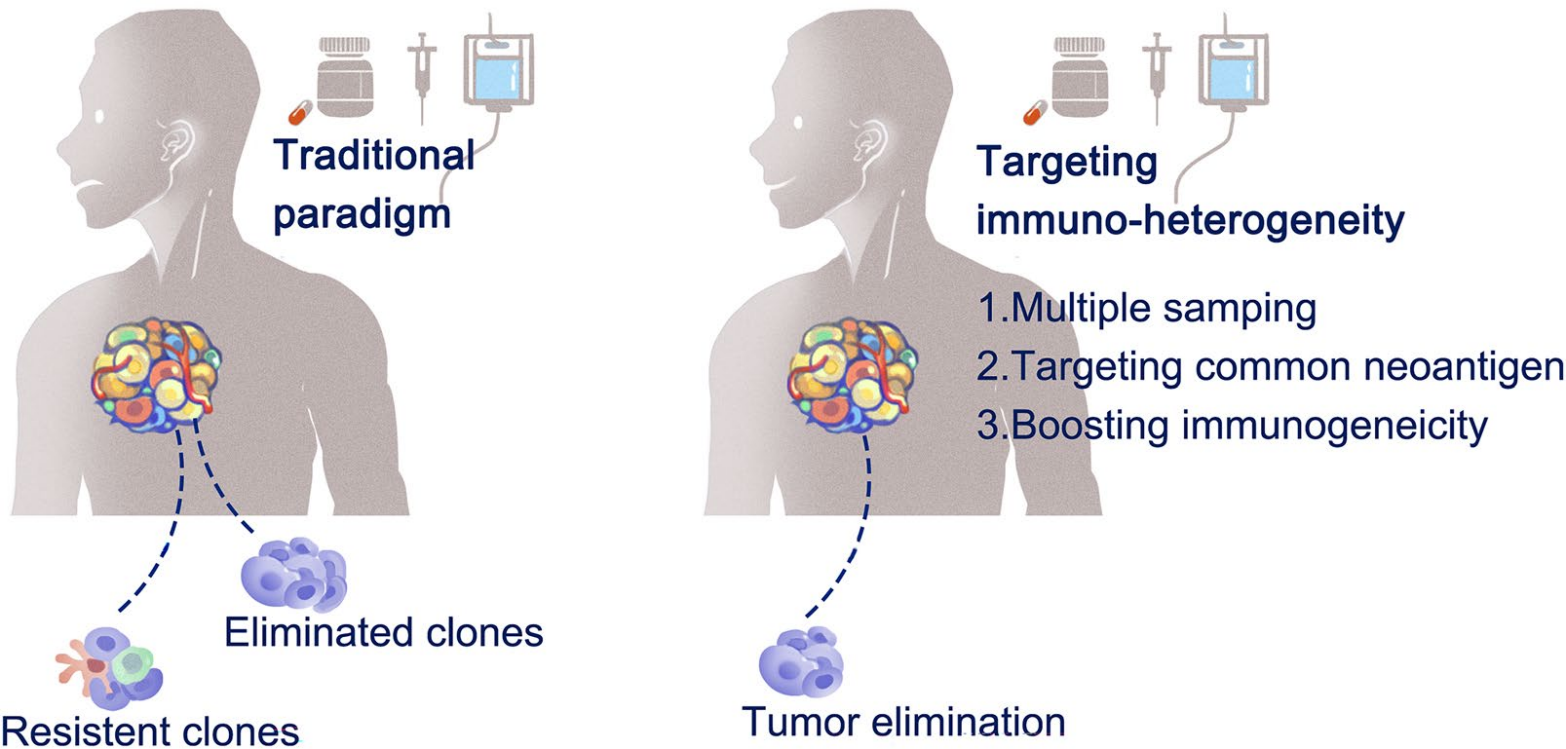
Strategies to overcome immunological heterogeneity-related resistance to therapies. Selective outgrowth of resistant clones to traditional therapeutic paradigms. Survived clones from initial anti-tumor treatment contribute to drug resistance. Alternatively, boosting immunogenic microenvironment with novel therapies contribute to overcome drug resistance

Multi-regional whole exome (or genome) sequencing has demonstrated that, not only in distinct anatomical locations (e.g., primary tumor vs. liver/brain metastasis), but also in different regions within the same tumor, there is substantial spatial heterogeneity in the genetic composition of tumor cells. Based on a longitudinal sampling strategy, sequencing studies have revealed that the genetic architecture of the same tumor is temporally heterogeneous [17]. In addition, tumor heterogeneity has a profound impact on the immune microenvironment. Various types of immune cell types show heterogeneous infiltration within tumors, including cytotoxic T lymphocytes (CTLs) [9, 18, 19], myeloid antigen-presentation cells [20], and cancer-associated fibroblasts (CAFs) [21]. Statistically, the magnitude of intratumoral genetic heterogeneity correlates with the heterogeneity of immune cell infiltration, implying the co-evolution of the tumor genetic architecture and immune microenvironment [22]. However, the crucial features that define tumor heterogeneity and its spatiotemporal evolution remain largely uncharacterized. Here, we summarize the driving force and composition of tumor heterogeneity (Figs. 1, 2) and its influence on tumor progression and response to immunotherapies, as well as strategies to overcome these unfavorable characteristics of evolutionary tumors (Fig. 3; Table 1).

**Table 1:** Therapeutic strategies to overcome heterogeneity of tumor immune microenvironment

Strategies	Regimen	Condition	Trial ID
Targeting common antigen	CD19 CAR-T	B-lineage NHL	NCT03029338, NCT02030834
	EGFR CAR-T	Lung, liver, stomach	NCT03179007, NCT03525782
	Mesothelin CAR-T	Ovarian, cervical, pancreatic, lung	NCT01583686
	Muc-1 CAR-T	Advanced solid tumors	NCT03179007, NCT03525782
	EpCAM CAR-T	Colon, pancreatic, prostate, gastric, liver	NCT03013712
	GD2 CAR-T	Glioblastoma	NCT04099797
	CEA CAR-T	Multiple tumor types	NCT02349724
	Glypican-3 CAR-T	Liver	NCT02932956
	DLL-3 CAR-T	Lung	NCT03392064
	Gp100 CAR-T	Melanoma	NCT03649529
	MAGE-A10 TCR-T	NSCLC	NCT02592577
	NY-ESO-1 TCR-T	Ovarian, melanoma, NSCLC	NCT01567891, NCT01350401, NCT02588612
	AFP TCR-T	Liver	NCT03132792
	MAGE-A3 TCR-T	Advanced solid tumors	NCT02153905
	WT1 TCR-T	Mesothelioma, NSCLC	NCT02408016
	HPV-16 E7 TCR-T	HPV-associated tumors	NCT02858310
	MART-1 TCR-T	Melanoma	NCT00706992
	EBV LMP2 TCR-T	NPC	NCT03925896
Prevailing with a multi-targeting strategy	Sequential CD19, CD20 CAR-T	B-lineage NHL	NCT03207178
	CD19-CD20 dual CAR-T	B-lineage NHL	NCT03398967, NCT03019055
	CD19-CD22 dual CAR-T	B-lineage NHL	NCT03593109, NCT-3468153, NCT03233854
Boosting immunogenic cell death and epitope spreading	T-VEC + pembrolizumab	Head and neck squamous cell carcinoma	NCT02626000
	HF-10 + ipilimumab	Melanoma	NCT02272855
	ONCOS-102 + cyclophosphamide	Advanced solid tumors	NCT01598129
	ONCOS-102 + pembrolizumab	Melanoma	NCT03003676
	OBP-301 + pembrolizumab	Advanced solid tumors	NCT03172819
	NeoVax + ipilimumab	Melanoma	NCT03929029
	PGV001 + Poly-ICLC	Advanced solid tumors	NCT02721043
	AutoSynVax + QS-21	Advanced solid tumors	NCT02992977
	mRNA-4157	Advanced solid tumors	NCT03313778
	Radiotherapy + ipilimumab	Melanoma	NCT01449279
	Radiotherapy + immature dendritic cells	Advanced solid tumors	NCT00278018
	Microwave ablation	Liver	NCT02851784

### Origin of heterogeneity of immune microenvironment

#### Origination from genetic instability

High-throughput sequencing approaches have long been used to depict the mutational spectrum and evolutionary trajectories of tumor cells. These studies delineate a broad scale of genetic tumoral heterogeneity in spatiotemporal dimensions [23, 24], including heterogeneous single-nucleotide variants, short indels, and copy number variants [25–27]. During tumor progression, genetic instability leads to the random generation of these alterations, either in the whole population (clonal tumor cells) or in a part of the population (subclonal tumor cells) [28]. In primary tumors, mutations in a driven gene usually deliver a survival advantage; therefore, these cells are more likely to occupy growth supplementation and develop to be a dominant clonal population [29]. In contrast, passenger mutations do not confer significant growth advantages in the course of tumor evolution [30]. They are considered to be the major origin of subclonal tumor cells. Therefore, genetic instability-originated clonal and subclonal tumor cells constitute the foundation of tumor evolution and spatiotemporal heterogeneity. At the same time, this genetic heterogeneity shapes the antigenic spectrum of tumors and ultimately contributes to the heterogeneity of the tumor immune microenvironment [31]. In particular, neoantigens, which are mainly derived from non-synonymous mutations and insertions/deletions, are the dominant driving force of divergent CD8^+^ T-cell specificity. Rather than the burden of neoantigen, a number of factors have been reported that could influence the quality of neoantigens, such as clonal fraction, similarity to self or known antigens, expression level, binding affinity of human leukocyte antigen, or the likelihood of neoantigen loss [32]. All these parameters primarily determine the neoantigenic immunogenicity, which mediates the CD8^+^ T cell response in the TME. A negative impact of clonal divergence has been observed on the sensitivity to therapeutics and disease outcomes in several studies [33–36]. With a bespoke sequencing strategy, TRACERx study revealed that the subclonal nature observed in phylogenetic ctDNA analysis was associated with disease relapse and metastasis among patients with non-small cell lung cancer (NSCLC) post primary surgery [34]. The clinical significance of clonal divergence has also been demonstrated in elderly patients with NSCLC. Gong et. al. found that elderly patients (> 60 years) were characterized by a loss of clonal neoantigens and decreased responsiveness to immune checkpoint blockade immunotherapies[37].

#### Origination from epigenetic modification

Accumulating evidence supports that epigenetic remodeling of tumor cells is also involved in the formation of a heterogeneous tumor immune microenvironment [38]. The mechanisms responsible for this modulation are mainly attributed to altered DNA modifications, modified chromatin accessibility, or modulation of gene expression at the post-transcriptional level such as that mediated by non-coding RNA interference. These epigenetic modifications fuel malignant progression of tumor cells and aid in shaping tumor immune microenvironment [39]. Along with strong chromosomal instability, longitudinal characterization of the methylation patterns among heterogeneous backgrounds identified progression potential in in situ lung carcinoma lesions [40]. In addition to methylation, various chromatin and epigenetic remodeling mechanisms confer a fitness advantage to tumor cells in response to the surrounding cues [38]. Typically, epigenetic modifications are conditionally reversible. In tumor cells, these modifications can be inherited by their offspring, and therefore, these cells display notable heterogeneity across spatial and longitudinal dimensions [41]. Epigenetic modifications can alter tumor progression and immunogenicity by affecting the accessibility and expression of immune-related elements [42]. Consistent with the observation of genetic instability, poor clinical outcomes have been associated with highly heterogeneous epigenetic modifications in multiple tumor types, including Ewing sarcoma [43], acute myeloid leukemia [44], and hepatocellular cell carcinoma [28].

#### Fitness to microenvironmental perturbations

Tumor cells are continuously exposed to extracellular microenvironmental perturbations. Growing evidence has shown that intracellular fitness can be initiated by external stresses, including the DNA damage response, unfolded protein response, and mitochondrial stress signaling [45]. Tumors display significant heterogeneity in histological and vascular architecture [46]. Regions proximal or distal to the vessels within the tumor are likely exposed to different oxygen supplementation [47]. Accordingly, the immune component could adapt to extrinsic stimuli based on oxygen tension, glucose availability, or oxidation pathway in a spatiotemporally, heterogeneous manner. Regardless of whether the immune component has adapted well (through survival or proliferation) under hypoxic conditions, almost all hypoxic responses are closely related to the reprogramming of the tumor immune microenvironment, which is mainly characterized by local switching of cell glycolytic metabolism, increased glucose consumption, increased pyruvate and lactate production, and acidification [48].

#### Response to anti-tumor treatment

During the course of treatment, tumor cells and all immune components in the microenvironment are either ‘punched’ by (e.g., hypo-fractionated radiotherapy), or under sustained exposure to (e.g., chemotherapy, target therapy, anti-angiogenic agents, or endocrine therapy) anti-tumor agents [49]. In response to these stressful agents, an adaptive mechanism initiates the tumor and immune compartment to establish a new homeostasis [50]. Due to the intrinsic heterogeneity of driver mutations or molecular characteristics, tumor cells are significantly different in their responsiveness to therapeutic treatment. Cytotoxic conditions imprint tumor and immune cells to undergo phenotype modification, cellular senescence, and even cell death. Local tumor clones that fail to survive the therapeutic agents release massive amounts of ATP through autophagy-mediated cell death [51]. These ATPs can facilitate chemotactic effects and provide an inflammatory space in the tumor [52]. In contrast, in the presence of extracellular nucleotidases, ATPs can be quickly digested to adenosine in the extracellular matrix, resulting in an inhibitory immune microenvironment [53]. For immune cell, the T cell phenotype changes dramatically in response to ICB. In patients with basal or squamous cell carcinoma, matched pre- and post-ICB tumor samples were subjected to scRNA-seq and scTCR-seq, and the results revealed a substantial replacement of pre-exited T cell pools accompanied by distinct T cell subset composition and cytokine production [54]. In patients with breast cancer and those receiving neoadjuvant therapy containing ICB, significant proliferation of CD8^+^ T cells was observed upon treatment in one-third of the patients. Additionally, the clonally expanded CD8^+^ T cells were characterized by pronounced expression of granzyme B, perforin, and CXCL13 [55]. These complex and dynamic interplays among therapeutic agents, spatialized tumor cell clones, and immune cell compartments significantly promote the formation of a spatiotemporal, heterogeneous, immune microenvironment.

### Heterogeneity of the tumor immune microenvironment

#### Spatial heterogeneity of the immune components

The characteristics of the tumor immune microenvironment are largely shaped by tumoral and non-tumoral components. Their localization or abundance/activity are spatially varied, including the surface expression of inhibitory immune checkpoints (such as well-known programmed death-ligand 1; PD-L1 [56]), or the secretion of immunosuppressive [57] or pro-inflammatory cytokines [58], the infiltration of immuno-suppressive or effector cells [59], and status of vasculature [60], or spatial distance to marginal region [61], or the distribution of metabolic nutrients [62]. These spatial variations also have a profound impact on the clinical prognosis and therapeutic response to treatment [63].

The phenotype of the intratumoral T cell compartment exhibits significant heterogeneity. Taking advantage of high-throughput sequencing approaches, T cells are usually characterized by different clonality, proliferative potential, differential stage, functional polarization, cytokine-secreting profiling, or metabolic environment. Genetic heterogeneity alone is weakly associated with intratumoral T cell phenotypes in patients with lung adenocarcinoma [64], and lung squamous cell carcinoma [65]. In another cohort investigating the determinants of local cytolytic activity in patients with NSCLC, both dominant T cell effector molecules (PRF1 and GZMB), and the expansion (diversity index) of whole T cell repertoire pools were almost independent of the mutational and neoantigen burden in their local niches [22]. Focusing on the propensity of T cell repertoires, expanded/proliferative T-cell receptors (TCRs) (TCR clones with high sequencing reads or frequency in the whole repertoire) can be further classified as common TCR clones (detected in all regions within the tumor), or regional clonal compartment (heterogeneously distributed) clones [66]. The number of common and regional TCR clones is positively correlated with the burden of common and regional non-synonymous mutations, demonstrating a regionally heterogeneous, antigen-driven proliferation of T cells. Theoretically, abundant neoantigens provide great potential for recognition by cognate T cells and subsequently lead to a higher magnitude of immune cell infiltration. However, there is evidence supporting the opposite of this scenario in a spatially heterogeneous manner. Local neoantigen burden was negatively associated with the infiltration of immune cells, including T cells, indicating immuno-pressure purification of neoantigen-coding mutations [65]. Similarly, in a cohort study of 212 samples from 38 patients with high-grade serous ovarian cancer, the epithelial CD8^+^ T cell compartment was negatively correlated with the genetic diversity of tumor cells, indicating an immunological depletion of antigenic subclones [67]. Notably, regulatory T cells (Tregs) also show significant intratumoral spatial heterogeneity and functional orientation [68].

The metabolic profile is a prominent modulator of the immune microenvironment, probably by influencing the proliferation potential and fitness of cancer cells to the environment. The heterogeneity of metabolic features appears to contribute to the heterogeneity of the tumor immune microenvironment. Malignant cells with high glycolytic activity can not only shift their metabolism pathway to anabolic reactions [69], but can also generate significantly increased amounts of immunosuppressive mediators such as lactate [70] and adenosine [71] to blunt immunosurveillance by cytotoxic cells. The heterogeneity of metabolic profiling has been documented in clonal and subclonal malignant melanocytes in a recent publication supporting their survival [72]. In addition, intratumoral heterogeneity in glycolysis was not revealed by imaging with a glucose fluorescence resonance energy transfer (FRET) biosensor in single-cell resolution with reversible transition [73], supporting the dynamic modulation of immune cell compartments.

In addition to the T cell subset in patients with lung cancer [66], intratumoral heterogeneity of many immune cell characteristics has also been identified among various tumor types, including NSCLC [74], gastric cancer [75], esophageal squamous cell carcinoma [76], non-Hodgkin lymphoma [77], glioma [78], and renal cell carcinoma [79]. In gastric cancer, macrophages with a CD68^+^CD163^+^CD206^+^ phenotype was found mainly located in stroma, and the CD68^+^IRF8^+^ macrophages were over-presented in the core region in comparison to the marginal zone [75]. In addition to immune cell populations, stromal cells also exhibit a high degree of spatial tropism in tumors. By comparing single cell sequencing data generated from tumoral core, middle, and tumoral edge regions in patients with NSCLC, seven distinct fibroblast subpopulations were identified. Three of them were found enriched in the core region of tumors, and two of them were enriched in tumoral edge [74]. These studies provide in-depth evidence of spatial heterogenous immune cell infiltration other than T cell compartments, such as dendritic cells, tumor-associated macrophages, and cancer-associated fibroblasts, potentially illustrating a dynamic balance between the malignant cells and immune cell compartment in the modulation of the anti-tumor immune response.

#### Temporal heterogeneity of the immune component

Notably, the tumor immune compartment is easily altered by environmental perturbation (genetic or non-genetic) and therefore determines the disease progression and response to anti-tumor treatments, together with the dynamic evolution of tumor cells themselves [80, 81]. During disease progression from non-invasive lesions to an invasive phenotype, a significant change in the composition of immune cell infiltration is revealed by RNA-Seq in patients with pancreatic ductal adenocarcinoma [82]. This temporal modification is generally made up by the decreased infiltration of CD8^+^ T cells and dendritic cells and is also frequently characterized by the aberrant accumulation or expansion of immunosuppressive cells, including Tregs, MDSCs, or CAFs. In a cohort study of the genetic and immunological propensities in patients with pre-invasive and early invasive lung adenocarcinoma, the authors observed the highest infiltration of CD8 T cells in the invasive adenocarcinoma, compared to the patients in the early stage of tumorigenesis (patients with adenocarcinoma in situ and minimally invasive adenocarcinoma). Subclonal mutations were preferentially identified in lesions positive for CD8 and PD-L1 expression [83].

Several studies have reported the migratory capacity of specific immune subsets and the replacement of infiltrating immune cells from adjacent tissue or peripheral circulation, which is extremely informative for the understanding of temporal immunological heterogeneity [84]. For instance, when tracking the transcriptional phenotypes and repertoire of T cells before and after anti-PD-1 treatment, pre-existing neoantigen-specific T cells showed under-estimated reinvigoration potential, and the T cell clones, which were bona fide responses to immunotherapy, migrated from a peripheral compartment of T cell clones and were highly distinct from their pre-treated counterparts [54].

Moreover, impaired cytolytic activity, constrained repertoire expansion and clonality, and progressive exhaustion of T and B cell compartments have been documented during disease progression in various tumor types including lung cancer [85], mouse model mammary carcinoma [86], melanoma [87], colon cancer [88], clear cell renal cell carcinoma [89], gastric adenocarcinoma [90], or in patients with intracranial metastatic lesions [91]. The emergence of immune-unfavorable regions or lesions in individual patients seems to be inversely proportional to disease control and survival prognosis, which further strengthens the importance of spatiotemporal heterogeneity for disease outcomes [92].

### Clinical Implications of tumor immune microenvironment

There is plenty of evidence that genetic heterogeneity increases the likelihood of malignant cells surviving in conventional chemotherapy, radiation therapy, and with targeted anti-cancer drugs. In addition, immune heterogeneity has a significant impact on the efficacy of immunotherapies, especially immune checkpoint blockade therapies.

#### Effect of tumor immune heterogeneity on survival outcomes

Almost all immunological prognostic or predictive biomarkers for patients with cancer were established based on assays of a single biopsied sample. However, heterogeneity is a significant obstacle to reproducibility across studies and attenuates their clinical practicability. For instance, in a cohort with pre-treated advanced melanoma (CA209-038 study), the cytolytic activity, a representative indicator of a ‘hot’ immune microenvironment, was increased during treatment and enriched in baseline tumor samples of anti-PD-1 responders [87]. Consistently, this molecular sign of a ‘hot’ tumor was also observed in several studies across various solid tumor types. In contrast, in another cohort of patients with metastatic melanoma, both pre-treated cytolytic activity score and interferon-γ pathway failed to associate with a favorable response to anti-PD-1 immunotherapy [93].

Despite being distributed in a spatially heterogeneous manner, neoantigens are usually randomly generated and universally present on the surface of recognized tumor cells. Therefore, theoretically, the heterogeneity of the immune microenvironment has a limited impact on the survival outcome of immunotherapy. However, ample evidence has demonstrated that the magnitude of heterogeneity in the tumor microenvironment, either genetic or immunologic, influences the efficacy of immunotherapies in patients with solid tumors. Sensitivity to immunotherapies can vary significantly depending on the heterogeneity of neoantigens and machinery of antigen presentation or cytotoxic signaling pathways [94, 95]. In a cohort of patients with metastatic NSCLC (discovery) or other solid tumor types (validation), higher intratumoral heterogeneity of mutations/neoantigens showed independent or joint (with mutational burden), predictive significance to poorer survival outcomes [96]. Experimental models also provide convincing evidence for the predictive value of immunological heterogeneity. With a novel ‘PresentER’ antigen presentation system, a mouse model demonstrated that immunogenic neoantigens do not always succeed in the elimination of tumor cells if only an extremely low portion of neoantigens is displayed on a single cell [97]. Tumors with more subclonal neoantigens are likely to diminish the responsiveness to checkpoint blockade therapies in comparison to homogeneous tumors [98, 99]. Notably, there is also evidence indicating a stronger immunogenicity of subclonal neoantigens [100], suggesting a complex role of clonal/subclonal neoantigens in determining the efficacy of immunotherapies.

The immunological environment of metastatic lesion-implanted organs contributes significantly to survival outcomes by compromising local or systemic anti-tumor immune responses. A recent study reported that liver metastases diminish immunotherapy efficacy systemically in patients and preclinical models. Patients with liver metastases have limited benefits from immunotherapy. In multiple mouse models, activated hepatic, antigen-specific Fas^+^ CD8^+^ T cells undergo apoptosis following their interaction with macrophages [101].

#### Heterogeneity of PD-L1 expression

Since the first evidence supporting PD-L1 protein expression (detected in tumor cells or immune cells by immunohistochemistry) and the efficacy of anti-PD-1 checkpoint blockade therapy, the PD-L1 level has been employed as an accompanying diagnosis to predict clinical response to ICB immunotherapies across various solid tumor types [102]. However, there is notable heterogeneous PD-L1 expression in intratumoral [22, 103] or inter-tumoral [104, 105] scales across both spatial and temporal dimensions [106, 107]. Upon evaluating PD-L1 expression in matched primary and brain-metastatic tumors derived from patients with NSCLC, Zhou et al. observed a significant discrepancy in PD-L1 expression between the two lesions [105]. PD-L1 expression was strongly induced by the interferon- γ (IFN-γ) signaling pathway, which is heterogeneously regulated in subclones harboring malfunctional JAK1/2 mutations [108, 109]. In addition, subclones with defects in antigen processing and presentation emerging in patients receiving ICB therapies have been associated with poor clinical outcomes in melanoma, lung cancer, and colorectal carcinoma. Such underlying heterogeneity may explain why a fraction of patients with PD-L1-positive tumors fail to respond, and some individuals with PD-L1-negative neoplasms respond well to ICB immunotherapies [110].

#### Heterogeneous response in TMB-high patients

Mutational burden, a reasonably approximate surrogate of neoantigen load, has been introduced to identify favorable responders to ICB immunotherapies in a variety of solid tumor types [111–113]. However, responsiveness to ICB treatment in TMB-high patients is highly heterogenous [114]. A considerable proportion of patients with low TMB could also benefit from ICB immunotherapies, and vice versa. For TMB-high patients who exhibit poor response to ICB treatment, defective/dysregulated antigen presentation machinery was considered to be the primary mechanism of resistance to immunotherapies [109, 115–117], especially the haplotype and regional expression of HLA [115, 118], and the expression of the B2M molecule [109]. In addition to this well-established view, based on an elaborate mouse model, Wolf et al. demonstrated that intratumoral heterogeneity promotes the aggressiveness of tumor cells, attenuating the provocation of anti-tumor immunity independent of mutational burden [99]. Furthermore, phylogenetic analysis identified that clonal heterogeneity, either measured by the number of clones composing tumor mass, or clonal divergence, had a profound impact on the survival outcomes of ICB treatment. Rather, the quantity of TMB, the mutated type of neoantigen-coding region [119] or the quality of TMB also influenced the predictive value of mutational load [120–122]. Other mutation spectrum-associated variables related to ITH include: (1) increased aneuploidy and consequent privilege cytotoxic activity [123], (2) epigenetic and chromatin alterations that regulate immune-related genes [124], and (3) metabolic reprogramming associated with insufficient antigen presentation and impaired anti-tumor immune response [125].

#### Heterogeneous response in deficient MMR patients

Patients with deficient MMR are extremely responsive to ICB treatment, which is largely attributed to elevated putative frameshift peptide neoantigens, and an improved immunogenic tumor microenvironment [126]. In a cohort of advanced dMMR patients across numerous solid tumor types, 53% of patients achieved radiological partial response and 21% patients achieved complete response [126]. This observation leads to the unprecedented approval of pembrolizumab as an optimal treatment for patients with MSI-H tumors, regardless of its histological sources [127]. However, only a few patients responded well to the ICB treatment. Tumor-cell intrinsic genotype, and extrinsic immunological circumstance of dMMR tumors can both modulate their efficacy and explain why primary and/or acquired resistance occurs during ICB immunotherapies. In dMMR tumors, the magnitude of immune cell infiltration genome instability seems to be largely heterogeneous, resulting in a discrete niche with limited immunogenicity and insufficient immune-mediated tumor control, which may lead to drug resistance. Similarly, despite the fact that shared immunogenic poly-epitopes have been reported in MSI-H tumors recently [128], the randomly generated mutational spectrum of dMMR tumors imparts higher plasticity in the neoantigen, substantially increasing the likelihood of the emergence of cytotoxic-resistant cancer cell clones despite a reinvigorated tumor immune microenvironment [129, 130]. Other ITH-related variables affecting the response of MSI-H tumors to ICI include their tendency to perform powerful immune editing and conversion to glycolysis profiles during development, which contributes substantially to immune escape.

### Strategies to overcome immune heterogeneity

#### Adoptive transferred cells targeting a common antigen

The presence of spatially different immunogenicity is the fundamental cause of the heterogeneous response to immunotherapy. A reasonable strategy to overcome this obstacle involves developing engineered cytotoxic cells that can target shared clonal neoantigens or homogeneously expressed tumor-associated antigens (TAA) across the entire tumor niche[131] (Fig. 3; Table 1). Firstly, a targetable epitope should be identified by immunohistochemistry profiling or by multi-regional high-throughput sequencing coupled with neoantigen prediction [32, 132]. Secondly, engineered cytotoxic cells with high affinity and specificity to this common target should be generated [133–135]. Treating B-lineage non-Hodgkin lymphoma (NHL) with chimeric antigen receptor T (CAR-T) is a typical application of this strategy [136]. Heterogeneous mutational spectrum and spatial distribution of several immune cell types have been observed in subsets of patients with diffuse large B-cell lymphoma [137, 138]. However, by targeting CD19, which was constitutively expressed by almost all malignant cells in DLBCL, more than half of the patients obtained durable long-term response from the CAR-T cell infusion; much higher than the objective response rate of anti-PD-1 monotherapies on DLBCL [139]. Additionally, CAR-T infusion can improve the therapeutic effect on solid tumors by synergistically combining with other treatment paradigms such as immune checkpoint blockade [140, 141], radiotherapy [142, 143], or tyrosine kinase inhibitors [144].

Overcoming heterogeneity by engineer-modified adoptive transferred cell therapy targeting common antigens remains unsatisfactory. It is effective in treating patients with solid tumors but requires considerable antigen density (currently, most tumors do not express a targetable, homogeneous clonal neoantigen) [145], and causes toxicity due to the target being expressed by normal tissues, cytokine release syndrome, or immune-mediated neurotoxicity [146]. During the course of disease progression or anti-tumor treatment, neoantigens and temporal neoantigen loss are major limitations of this strategy. This limitation may be partially overcome by targeting alternative neoantigens that are generated from prevalent oncogenic mutations or infusions. Because of their indispensable biological role in maintaining the survival and progression of tumors, these targets are usually constitutively expressed on the surface of malignant cells. Other potential obstacles include the emergence of T cell exhaustion, an immunosuppressive or excluded immune microenvironment, or intrinsic resistance signaling of tumor cells.

#### Prevailing with a multi-targeting strategy

Considering the temporal loss of shared targetable antigens, it is reasonable to overcome immunogenic heterogeneity by targeting multiple antigens simultaneously with modified, engineered, adoptive transferred cell therapy (Fig. 3; Table 1). This strategy maximizes the likelihood of preventing immune evasion upon antigen loss due to heterogeneous immunogenicity in solid tumors. In the case of CAR-T-treated B-cell NHL, CD19 evasion in subclonal tumor cells has been confirmed as a mechanism of resistance to anti-CD19 CAR-T therapies [147]. Therefore, it is reasonable to cover these evaded subclones with alternative, broadly targetable epitopes as complementary insurance for effective clearance of tumor cells as much as possible. These dual-target strategies can be achieved by (even more than) two types of CAR-T cells targeting distinct epitopes separately, or by a single modified CAR-T product that targets multiple epitopes simultaneously. In line with this principle, various targetable TAAs have been proposed to overcome heterogeneous antigenicity, such as CD20 [148, 149], CD22 [150], and CD79b [151]. The performance of anti-CD38 CAR-T cells has been tested in patients with B-cell NHL and relapse from anti-CD19/CD22, bi-specific CAR-T cell therapies [152].

#### Boosting immunogenic cell death and epitope spreading

Tumor vaccination is a promising therapeutic strategy in cancer immunotherapy, not only because of its ability to provoke an inflammatory circumstance by delivering highly immunogenic antigens, but also because of their potential to broaden and diversify the antigenic spectrum by fostering epitope spreading [153] (Fig. 3; Table 1). Epitope spreading is a dynamic process that refers to the diversification of epitope specificity from the initially targeted trigger epitope to a broader immunogenic spectrum that includes cryptic epitopes or epitopes with suboptimal affinity to cognate T cell repertoire clones. Therefore, it is reasonable to expect a diversified T cell response with more comprehensive coverage of the whole tumor regardless of their spatial heterogeneity, especially in combination with other approaches aimed at remodeling the immunosuppressive microenvironment. Facilitating with breakthroughs in the functional identification of personal neoantigens [32, 154], a robust neoantigen-specific T cell response was continuously detected in the long-term clinical course in patients who were treated with synthesized neoantigens [155, 156]. Along with the persistence of neoantigen-specific T cell clones, a diversified T cell repertoire with broader specification (emergence of additional T cell clones targeting distinct tumor-associated antigens) was also observed following the vaccination, providing a greater likelihood of full coverage of the tumor mass with heterogeneous antigenicity. In addition to directly delivering synthesized peptides, RNA vaccines coding for personalized neoantigens [157], or dendritic cell-loaded neoantigens [158], have also been successful in mobilizing an effective and sustained antitumoral immunity with a broad T cell specificity.

Another strategy to overcome the heterogeneity of the immune microenvironment relies on the direct oncolysis of malignant cells with engineered viruses [159] which strongly promotes immunogenic cell death with the release of abundant amounts of immuno-active elements, including cryptic tumor-associated antigens, danger signals, cytokines, and chemokines [160]. This accompanied paracrine action is critically important for activating unselective T cell cytotoxicity in spatially surrounding intratumoral loci, particularly for the loci with relatively less immunogenicity. In general, the efficacy of oncolytic viruses as monotherapy is unsatisfactory. However, coupled with the release of immuno-active components, oncolytic viruses have shown the potential to facilitate subsequent (or concurrently delivered) immunotherapies by establishing an immune microenvironment with a lower threshold to initiate effective T cell recognition against neoantigens [161, 162]. In addition to oncolytic viruses, various approaches have been introduced to provoke an inflammatory immune microenvironment through a soluble component-mediated mechanism, such as novel pharmaceutical agents [163, 164], tyrosine kinase inhibitors [165], cationic ampholytic peptides [166], microwave [167], and radiotherapy [168, 169] which is highly effective in inducing inflammatory immunological status [170] and easily irradiates the tumor mass with an appropriate spatial coverage. All of this evidence underscores the critical issue that robust therapeutic responses generally involve the remodeling of the entire immune microenvironment towards an immuno-active, homogeneous one.

## Conclusions

Tumorigenesis represents an integrated and accumulated dysregulation of a series of genetic and non-genetic processes. Due to the intrinsic genetic instability of the tumor genome, a large portion of tumorigenic events inevitably occur in a random manner during disease progression. These random events create an essential substrate for the development of a heterogeneous immune microenvironment, either in the spatial or temporal dimensions. Additionally, competition for metabolites and nutrients, therapeutic pressures, or the evolution of key oncogenes continuously remodel the immune microenvironment. This ultimately creates the opportunity for malignant cells to escape from immunosurveillance, eventually resulting in disease progression and metastasis. This immunological heterogeneity also underlies the poor performance of single biopsy-based predictive biomarkers and resistance to immunotherapies. Even in an ideal scenario of single oncogene-driven tumors, an expected perturbation introduced by a highly specific target therapy could further break the balance between the tumoral component and immune system within tumors, thereby increasing the heterogeneity of the immune microenvironment. Treatment-related survival pressure also accelerates the evolution of malignant cells, leading to exacerbated complexity of the oncogene spectrum, as well as the resulting immune compartment. Conclusively, regardless of the mechanism of the paradigm of anti-tumor treatment or the detailed process of tumor evolution, the complication of the immune microenvironment and resistance to immunotherapies almost definitely occurs in patients with solid tumors.

The co-evolution of tumors and immune compartments supports the temporal heterogeneity of the immune microenvironment, particularly under the pressure of therapeutic approaches. T cell (or other immune cells) clonal dynamics in longitudinally sampled tumor tissue has substantial potential to guide personalized immunotherapies, thereby addressing the challenge of temporal heterogeneity of the immune microenvironment. However, the obstacle to performing longitudinal tissue biopsies at regular intervals or sampling multiple tumor tissues simultaneously suggests that this strategy should ideally be combined with the use of a non-invasive, liquid biopsy approach. A qualified and cost-effective liquid biopsy pipeline enables a more regular and frequent immune surveillance in clinical practice and facilitates interventions that improve patient outcomes. The association between liquid biomarkers and intra-/intertumoral immunological heterogeneity remains largely unknown. Therefore, trials designed to investigate the benefits of liquid biopsies for inferring the therapeutic implications are urgently needed. However, the sensitivity and accuracy of the current liquid biopsy assays are far from satisfactory. Nevertheless, this approach might eventually be an ideal platform for determining the full extent of heterogeneity of the tumor immune microenvironment, tracking the emergence of resistance to immunotherapy, and identifying optimal therapies that can precede and overcome heterogeneity.

Finally, considering the instability of the tumor genome and the endless development of heterogeneity, it is imperative to concentrate on the lessons learned from models of heterogeneous tumors, either mouse model [99] or in silico estimated [171]. A sophisticated, controlled model enables us to precisely understand the mechanisms underlying the modulation of the anti-tumor immune response to heterogeneity. Therapeutic approaches should account not only for the oncogenic target or representative immune checkpoint but also for the heterogeneity and responsiveness of the immune microenvironment. Navigating the spatiotemporal interaction between tumorous and immunological compartments is critical in informing effective and prolonged responses to immunotherapies.

## Data Availability

Not applicable.
